# STAMP/STOMP Audit: Psychotropic Medication Prescribing and Physical Health Monitoring, for Children and Young People in the Leeds Community Healthcare (LCH) Learning Disability Clinic

**DOI:** 10.1192/bjo.2025.10540

**Published:** 2025-06-20

**Authors:** Julius Akhetuamhen, Eleanor Morris

**Affiliations:** 1 Leeds and York Partnership NHS Foundation Trust, Leeds, United Kingdom; 2 South West Yorkshire Partnership NHS Foundation Trust, Wakefield, United Kingdom

## Abstract

**Aims:** This audit explores adherence of the Leeds Community Healthcare (LCH) Learning Disability Clinic to the STAMP/STOMP guidelines. This is to ensure that children with moderate to severe learning disability or autism are not overmedicated with psychotropic medications and are aware of their right to an annual health check.

**Methods:** This audit took place in 2 cycles: JA in November 2023 and EM in July 2024. On both occasions, 30 clinic patients were randomly selected. Data was collected from SystmOne and Leeds Care Record, to ascertain:

The number of children who had their Annual Health checks in the previous year.

Whether the drug names and dosages had been identified in case notes.

Whether indications for psychotropic treatment were documented.

If the drug was within British National Formulary limits.

Whether there was a discussion of side-effects at initiation and follow-up.

In cycle 2, EM set criteria for “enquiry for side-effects at follow-up”. This was interpreted as a relevant medical appointment in the past 6 months or since a change in dose. In addition, the age of the child was accounted for in the second cycle, as only children >14 years were advised to have an annual physical health review.

**Results:** Children receiving an annual health check: 70% (cycle 1)/80% (cycle 2).

Drug names and dosages have been documented: 100% (cycles 1 and 2).

Indication for psychotropic medications has been documented: 85% (cycle 1)/93% (cycle 2).

Discussion of side-effects at initiation has been documented: 73% (cycle 1)/38% (cycle 2).

Enquiry for side-effects at follow-up: 77% (cycle 1)/54% (cycle 2).

**Conclusion:** Medications and dosage were consistently documented across both Audits. In cases where only melatonin or ADHD medication is prescribed, it was more common to find that discussion of adverse effects, and specific impacts on sleep duration and latency were not documented. On an ongoing basis, team members must ensure that patients over the age of 14 (and their families) are aware of their right to an annual physical health check.

One limitation of this study is that ‘discussion of side-effects’ does not clarify the extent and frequency to which these should be discussed. This may account for the large difference between audit cycles. In addition, the spread of information between systems may have increased the possibility of information being lost or overlooked.

